# Effects of Pancreatic Duct Stenting on Nutrition and Pancreatic Function in Unresectable Pancreatic Cancer: A Pilot Study

**DOI:** 10.1002/jgh3.70358

**Published:** 2026-03-07

**Authors:** Ko Watsuji, Kenji Ikezawa, Yugo Kai, Ryoji Takada, Masaki Kawabata, Hiroki Kishimoto, Kana Hosokawa, Yusuke Seiki, Kazuhiro Kozumi, Makiko Urabe, Kaori Mukai, Tasuku Nakabori, Kazuyoshi Ohkawa

**Affiliations:** ^1^ Department of Hepatobiliary and Pancreatic Oncology Osaka International Cancer Institute Osaka Japan; ^2^ Department of Gastroenterology and Hepatology Kansai Rosai Hospital Hyogo Japan

**Keywords:** ERCP, obstructive pancreatitis, pancreatic cancer, pancreatic duct stenting, pancreatic exocrine insufficiency

## Abstract

**Aims:**

Malnutrition due to pancreatic enzyme insufficiency and cancer‐related systemic effects is common in advanced pancreatic cancer and worsens survival. Pancreatic duct stenosis can lead to obstructive pancreatitis, enzyme elevation, and nutritional decline. In this study, we aimed to evaluate the impact of transpapillary pancreatic duct stenting in patients with unresectable pancreatic cancer.

**Methods and Results:**

This retrospective study included patients with obstructive pancreatitis or pancreatic enzyme elevation due to pancreatic duct stenosis caused by unresectable pancreatic cancer who underwent transpapillary pancreatic duct stenting at Osaka International Cancer Center (November 2020–July 2022). Patients were selected based on specific criteria, including available computed tomography scans 3 months post‐stenting and continued chemotherapy. Nutritional, clinical, and pancreatic structural changes were assessed 3 months after stent placement. The Wilcoxon signed‐rank test was used for statistical analysis. All 10 patients achieved clinical success without complications. The main pancreatic duct diameter significantly decreased at the body (5.8 to 3.6 mm; *p* = 0.004) and tail (3.8 to 2.0 mm; *p* = 0.025); however, pancreatic parenchymal thickness remained stable. Nutritional markers showed a trend toward improvement, with a significant gain in body weight from 47.2 to 48.6 kg (*p* = 0.048).

**Conclusion:**

Pancreatic duct stenting may be associated with improvements in nutritional status and the maintenance of pancreatic structure in patients with unresectable pancreatic cancer, suggesting a potential role in managing pancreatic enzyme insufficiency and malnutrition.

## 1 Introduction

Despite advances in treatment, pancreatic cancer has a poor prognosis, particularly in unresectable cases [1, 2]. Malnutrition is common in these patients due to pancreatic enzyme insufficiency and the systemic effects of malignancy [3, 4, 5], which contribute to physical decline and reduced chemotherapy tolerance, further worsening survival outcomes [6, 7, 8].

Pancreatic duct stenosis in pancreatic cancer can lead to obstructive pancreatitis and elevated pancreatic enzyme levels, impairing pancreatic function and exacerbating malnutrition [9, 10]. Pancreatic duct stenting is an established intervention for relieving ductal hypertension, managing symptoms of chronic pancreatitis, and preventing post‐endoscopic retrograde cholangiopancreatography (ERCP) pancreatitis [11, 12, 13]. However, its effectiveness in malignant pancreatic stenosis remains unclear.

This study aimed to evaluate the impact of transpapillary pancreatic duct stenting on pancreatic function and nutritional status in patients with unresectable pancreatic cancer. A retrospective analysis assessed clinical, structural, and nutritional outcomes 3 months after stent placement.

## 2 Methods

### 2.1 Patients

**FIGURE 1:**
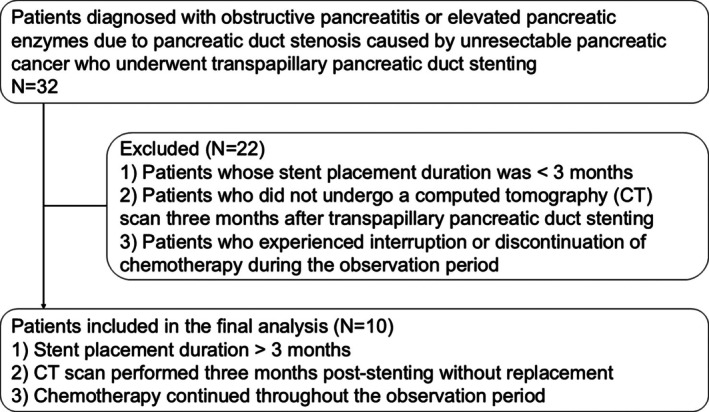
Flowchart of patient selection.

We retrospectively reviewed the medical records of 32 patients diagnosed with obstructive pancreatitis or elevated pancreatic enzymes due to pancreatic duct stenosis from unresectable pancreatic cancer who underwent pancreatic duct stenting at the Osaka International Cancer Center between November 2020 and July 2022. Patients were excluded if they had a stent placement duration of less than 3 months, did not undergo a computed tomography (CT) imaging 3 months after transpapillary pancreatic duct stenting, or experienced interruption or discontinuation of chemotherapy during the observation period. Ultimately, 10 patients met the following criteria and were therefore included in the analysis: (1) stent placement duration exceeded 3 months, (2) a CT scan was performed 3 months post‐stenting without replacement, and (3) chemotherapy continued throughout the observation period (Figure 1). These criteria were selected to assess the long‐term effects of stenting on pancreatic duct stenosis. Additionally, extended follow‐up data up to 6 months were collected when available. Diagnoses of pancreatic cancer were established through pathological confirmation, primarily using endoscopic ultrasound‐guided tissue acquisition. Unresectable pancreatic cancer was diagnosed according to the National Comprehensive Cancer Network (NCCN) guidelines [14]. Clinical staging and resectability were evaluated by a multidisciplinary cancer board comprising surgeons, oncologists, and radiologists, particularly in cases requiring further discussion. The study was approved by the Institutional Review Board of the Osaka International Cancer Institute (18050–7).

### 2.2 Endoscopic Procedures

ERCP was performed under fluoroscopic guidance using a side‐view endoscope (TJF260V; Olympus, Tokyo, Japan) with sedation (midazolam) and analgesics (pethidine). All procedures were conducted by endoscopists with at least 5 years of experience. Pancreatic duct cannulation was attempted using a PR‐104Q‐1 (Olympus) or MTW cannula (Wesel, Germany) via contrast or guidewire techniques. Following successful cannulation, pancreatography was performed to evaluate the location and characteristics of the stenosis. A guidewire and a 5 Fr Advanix Pancreatic Stent (Boston Scientific; Natick, MA, USA) or a 7 Fr Pancreatic Stent (Olympus) were placed across the stenotic region. The stent length was determined based on stenosis location and severity as assessed by pancreatography. Complications were monitored for at least 3 months in all patients through clinical evaluation, laboratory tests, and CT imaging, with extended follow‐up up to 6 months when available.

### 2.3 Definitions

Clinical data were obtained from the medical records. Blood tests, including peripheral and biochemical analysis, along with weight measurements, were conducted before and after stenting. Follow‐up tests were performed 3 months post‐stenting, within a two‐week window. A contrast‐enhanced CT scan was conducted before and 3 months after stent placement. The thickness of the pancreatic parenchyma was measured as the maximum thickness at the pancreatic body or tail minus the diameter of the main pancreatic duct (MPD) on coronal CT images. MPD and parenchymal thickness measurements were taken at the level of the abdominal aorta (pancreatic body) and the left kidney (pancreatic tail).

### 2.4 Obstructive Pancreatitis and Pancreatic Enzyme Elevation

Acute pancreatitis was diagnosed based on at least two of the following criteria: (1) acute upper abdominal pain and tenderness; (2) elevated blood or urinary pancreatic enzyme levels; and (3) abnormal pancreatic imaging finding (e.g., swelling, peri‐inflammation, or edema) on ultrasound, CT, or magnetic resonance imaging (MRI) [15]. Obstructive pancreatitis was defined as acute pancreatitis caused by pancreatic duct stenosis due to pancreatic cancer with an indication for stent placement. Persistent pancreatic enzyme elevation in the presence of stenosis, even without pancreatitis symptoms, was classified as a high‐risk condition for the development of obstructive pancreatitis and was thus deemed an indication for stenting. Clinical success was defined as resolving obstructive pancreatitis, or significantly reducing pancreatic enzyme levels.

### 2.5 Statistical Analysis

Continuous variables before and 3 months after stenting were compared using the Wilcoxon signed‐rank test, chosen for its suitability in analyzing paired nonparametric data from a small sample size. Statistical significance was set at *p* < 0.05. All statistical analyses were conducted using EZR (Saitama Medical Center, Jichi Medical University, Saitama, Japan), a graphical user interface for R version ver. 1.68 (The R Foundation for Statistical Computing, Vienna, Austria).

## 3 Results

### 3.1 Patient Characteristics

Table 1 summarizes the characteristics of the 10 patients included in the analysis. Four patients (40%) were male, with a median age of 60.5 years (range: 53–81). All cases of pancreatic stenosis were caused by primary pancreatic cancer, with a median tumor diameter of 28 mm (range: 20–60). Regarding disease stage, four patients had unresectable locally advanced pancreatic cancer, whereas six had unresectable metastatic pancreatic cancer. Nine patients (90%) had stenosis at the pancreatic head, while one (10%) had stenosis at the pancreatic body and tail. Eight patients (80%) underwent concurrent endoscopic biliary drainage. None of the patients in this study received pancreatic enzyme replacement therapy, as no overt symptoms warranted its use.

**TABLE 1:** Patient characteristics (*N* = 10).

Characteristic	*N* = 10
Sex	
Male	4 (40%)
Female	6 (60%)
Median age (years)	60.5 years (range: 53–81)
Median tumor size (mm)	28 mm (range: 20–60)
Stage	
Unresectable locally advanced	4 (40%)
Metastatic	6 (60%)
Location of the pancreatic duct obstruction	
Head	9 (90%)
Body/tail	1 (10%)
Indications for pancreatic duct drainage	
Obstructive pancreatitis	6 (60%)
Pancreatic enzyme elevation	4 (40%)
Concurrent biliary stenting	
Yes	8 (80%)
No	2 (20%)

### 3.2 Stent Placement and Complications

Of the 10 patients, nine received a 5 Fr pancreatic ductal stent, and one received a 7 Fr stent. Clinical success was achieved in all cases, with no complications reported.

### 3.3 Pancreatic Duct Stenting Outcomes: Structural and Nutritional Changes

The MPD diameter decreased significantly 3 months after stent placement compared to its preplacement measurement. At the pancreatic body, the ductal diameter decreased from 5.8 to 3.6 mm (*p* = 0.004), and at the pancreatic tail, from 3.8 to 2.0 mm (*p* = 0.025) (Table 2).

**TABLE 2:** Comparison of pancreatic function and nutritional status between before and 3 months after pancreatic stent placement (*N* = 10).

Parameters	Before stent placement Median (range)	Three months after stent placement Median (range)	*p*
Albumin (g/dL)	3.9 (2.1–4.4)	4.1 (2.5–4.4)	0.236
HbA1c (%)	5.9 (5.0–8.7)	5.8 (5.1–8.9)	0.285
Body weight (kg)	47.2 (40.8–81.6)	48.6 (40.5–91.4)	0.048*
Amylase (U/L)	342 (121–430)	91 (60–158)	0.002*
Maximum main pancreatic duct diameter (body) (mm)	5.8 (3.0–8.0)	3.6 (1.8–4.2)	0.004*
Maximum main pancreatic duct diameter (tail) (mm)	3.8 (1.7–5.9)	2.0 (0.9–4.1)	0.025*
Pancreatic thickness (body) (mm)	8.6 (6.6–19.5)	9.2 (5.6–19.5)	0.557
Pancreatic thickness (pancreas tail) (mm)	12.8 (6.5–20.2)	13.4 (8.2–27.0)	0.275

*Note: p*‐values < 0.05 are considered statistically significant and are marked with an asterisk (*).

Pancreatic parenchymal thickness remained stable, with no significant differences observed. At the pancreatic body, thickness increased slightly from 8.6 to 9.3 mm (*p* = 0.557), and at the pancreatic tail, from 12.8 to 13.4 mm (*p* = 0.275).

**FIGURE 2:**
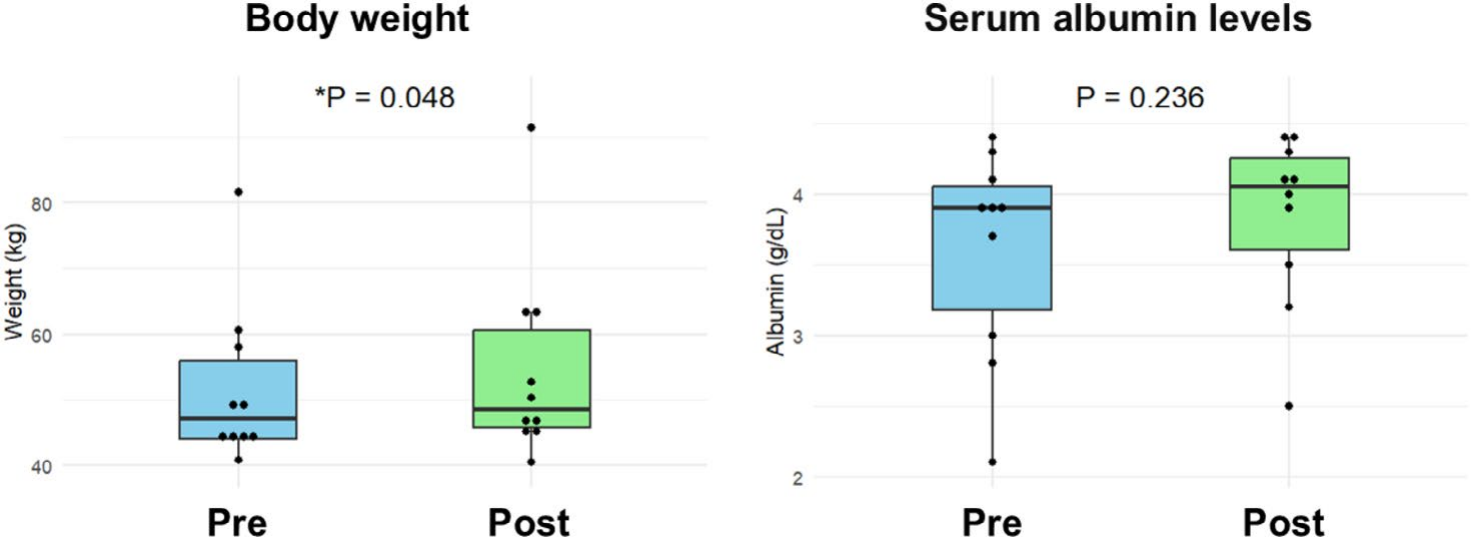
Changes in body weight and serum albumin levels before and 3 months after pancreatic duct stenting.

Nutritional markers showed a trend toward improvement. Body weight significantly increased from 47.2 to 48.6 kg (*p* = 0.048), while serum albumin levels also increased from 3.9 to 4.1 g/dL, although this change was not statistically significant (*p* = 0.236) (Figure 2). These results suggest that pancreatic duct stenting may be associated with potential nutritional benefits.

Furthermore, six patients were followed for up to 6 months without experiencing any complications or requiring stent exchange. During this extended follow‐up period, although the changes were not statistically significant, there was a continued trend toward increased albumin levels (3.4–3.7 g/dL) and body weight (48.8–50.5 kg). Of the remaining four patients, two were lost to follow‐up after 3 months due to disease progression, and the other two underwent stent exchange. There were no pancreatic duct stent‐related complications observed in any of these cases.

## 4 Discussion

This study demonstrated that transpapillary pancreatic duct stenting in patients with obstructive pancreatitis or elevated pancreatic enzymes due to pancreatic duct stenosis from unresectable pancreatic cancer was associated with a trend toward improved nutritional markers. These findings suggest that stenting may relieve pancreatic duct obstruction and help to preserve pancreatic function and nutritional status by preventing pancreatic atrophy and correcting exocrine insufficiency.

Cancer‐related weight loss and malnutrition are major clinical concerns [16, 17, 18], affecting 42% of patients with pancreatic cancer, with weight loss exceeding 10% within 6 months of diagnosis [19]. Various nutritional interventions have been studied, such as dietary counseling, home‐based parenteral nutrition, and selective ghrelin receptor agonists [20, 21, 22, 23]. Pancreatic enzyme replacement therapy has been shown to alleviate weight loss and symptoms like diarrhea, steatorrhea, abdominal bloating, and excessive gas [24, 25]. Since pancreatic atrophy and exocrine dysfunction often develop secondary to main pancreatic duct obstruction [26], pancreatic duct drainage may help maintain pancreatic function and reduce nutritional deficiencies [27]. However, research on the role of stenting in patients with advanced pancreatic cancer is limited.

Domínguez‐Muñoz et al. demonstrated that transpapillary pancreatic drainage led to remarkable improvements in exocrine pancreatic function, along with elevated levels of prealbumin and retinol‐binding protein, as early as 2 weeks after the procedure in patients with unresectable pancreatic cancer [28]. In contrast, our study observed a trend toward sustained nutritional improvement over a 3‐month period after pancreatic duct stenting. This suggests that while early responses may occur within weeks, as reported by Domínguez‐Muñoz et al. longer‐term nutritional benefits, including weight gain and maintenance of pancreatic parenchymal thickness, may be observed with continued decompression of the pancreatic duct. These findings indicate that pancreatic duct stenting may contribute not only to early but also long‐term maintenance of nutritional status in patients with unresectable pancreatic cancer.

Our study also showed that pancreatic parenchymal thickness remained stable for 3 months following stenting. Pre‐ and postoperative CT imaging studies have shown that pancreatic atrophy correlates with exocrine insufficiency, with postoperative atrophy serving as a predictor of pancreatic dysfunction [29]. Volumetric assessment of the pancreatic parenchyma has also been linked to exocrine function [30]. In the present study, the preservation of pancreatic parenchymal thickness suggests a possible role for stenting in preventing pancreatic atrophy, which may contribute to better exocrine function and long‐term nutritional maintenance. These results indicate that pancreatic duct stenting could play a role in facilitating pancreatic drainage and preserving structural integrity and function, potentially aiding in exocrine insufficiency management in patients with advanced pancreatic cancer.

Although endoscopic pancreatic stenting is primarily used for chronic pancreatitis pain relief, managing pancreatic duct disruptions, and reducing post‐ERCP pancreatitis [31, 32], its role in malignant stenosis is less understood. Reported complications include acute pancreatitis, stent occlusion, and stent migration, with an overall complication rate of 7.85% [33], with half of all complications occurring within 30 days [34]. Stent migration can lead to severe outcomes, such as pancreatic parenchymal injury or splenic artery perforation [35, 36, 37], while stent fracture can pose significant retrieval challenges [38, 39, 40]. Siddappa et al. summarized data from 12 studies involving 192 patients with pancreatic cancer presenting with abdominal pain who underwent pancreatic duct decompression, reporting a technical success rate of 87%. The complication rates were relatively low, including pancreatitis (0.6%), bleeding (1.8%), stent migration (3.6%), and stent occlusion (3.0%) [41]. A recent randomized controlled trial by Sun et al. assigned 80 patients with unresectable pancreatic cancer and biliary obstruction to either biliary stenting alone or combined biliary and pancreatic duct stenting, revealing that the addition of pancreatic duct stenting did not increase complication rates [42]. In our present study, although limited by a small sample size of 10 patients, no stent‐related complications were observed. Nevertheless, given the potential for serious adverse events, careful patient selection and close monitoring for complications are crucial when considering pancreatic duct stenting in patients with pancreatic cancer.

These results should not be generalized to all patients with pancreatic cancer. In cases of obstructive jaundice, simultaneous pancreatic duct drainage may be considered alongside biliary drainage [27]. Nevertheless, carefully evaluating the risks and benefits of pancreatic stent placement remains essential. Clinical decisions regarding endoscopic pancreatic duct placement should be individualized based on patient circumstances and clinical needs.

This study has some limitations. First, as a single‐center retrospective pilot study with a relatively small sample size, the findings are exploratory in nature and may have limited generalizability. Second, due to the absence of a comparable cohort of patients who met similar inclusion criteria but did not undergo stenting, direct comparisons to alternative treatments or the natural course of pancreatic duct stenosis in advanced pancreatic cancer could not be assessed.

In conclusion, transpapillary pancreatic duct stenting appeared to be associated with trends toward improved nutritional status and the preservation of pancreatic parenchymal thickness in patients with obstructive pancreatitis or elevated pancreatic enzymes due to pancreatic duct stenosis in unresectable pancreatic cancer. These findings indicate that stenting may mitigate pancreatic exocrine insufficiency and its impact on malnutrition in this patient population. However, given the retrospective design and small cohort size, these findings should be interpreted with caution, and larger prospective studies are warranted to confirm the generalizability and long‐term efficacy of pancreatic duct stenting in this setting.

## Funding

The authors have nothing to report.

## Ethics Statement

This study was approved by the Institutional Review Board of the Osaka International Cancer Institute (Approval No.: 18050–7) and was conducted following the ethical principles of the 1964 Declaration of Helsinki and its subsequent amendments.

## Consent

Informed consent to participate in the study was waived, in accordance with the Japanese government's Ethical Guidelines for Medical and Health Research Involving Human Subjects, which allow an opt‐out approach for studies based on existing, anonymized information that do not include human biological specimens.

## Conflicts of Interest

The authors declare no conflicts of interest.

## Data Availability

The data that support the findings of this study are available from the corresponding author upon reasonable request.
